# Special Issue “The Angiotensin in Human Health and Diseases”

**DOI:** 10.3390/ijms262411940

**Published:** 2025-12-11

**Authors:** Helmy M. Siragy

**Affiliations:** Department of Medicine, School of Medicine, University of Virginia, Charlottesville, VA 22903, USA; hms7a@uvahealth.org

The renin–angiotensin–aldosterone system (RAAS) plays an important role in body fluid and electrolyte homeostasis and blood pressure regulation. In addition to its circulating effects, the RAAS has a local tissue component that regulates cellular growth, matrix formation, and cardiovascular, renal, and metabolic functions [1]. Excess RAAS activity can produce specific pleiotropic effects [2] that contribute to tissue inflammation [3], fibrosis [4], immune cell reprogramming [3], and metabolic dysfunction [5].

This Special Issue focuses on the evolving knowledge involving RAAS activities in human health and disease. The collective findings from recent papers published in the current Special Issue of the International Journal of Molecular Sciences provide a cohesive and compelling framework: RAAS contributes a common pathway connecting vascular inflammation, adipose dysfunction, renal injury, hepatic fibrosis, and the developmental origins of cardiometabolic disease (Figure 1).

In the first contribution to this Special Issue, Zhang et al. (contribution 1) demonstrate that aldosterone promotes phenotypic switching of vascular smooth muscle cells (VSMCs) into macrophage-like cells, thereby directly contributing to inflammatory vascular lesions. This study underscores the ability of RAAS components to induce cellular plasticity, immune cell mimicry, and pro-inflammatory cytokine release, highlighting a previously underappreciated mechanistic link between hormonal signaling and vascular inflammation [6,7].

The second contribution to this Special Issue elucidates the role of aminopeptidase A (APA) in the central and peripheral regulation of blood pressure through the conversion of angiotensin II to angiotensin III (contribution 2). These findings clarify the enzymatic complexity within RAAS and identify APA as a potential therapeutic target for selectively modulating central hypertension, without the systemic hypotension commonly associated with traditional RAAS blockade.

The third contribution to this Special Issue reveals that diabetic nephropathy is characterized by augmented intrarenal and urinary angiotensinogen, driving intrarenal RAAS activation and renal injury (contribution 3). Isoflavone treatment mitigated this effect, suggesting a promising nutritional or pharmacological strategy to modulate organ-specific RAAS activity.

In the fourth contribution, Mkhize et al. identify elevated plasma ACE and angiotensin II levels in prediabetic individuals, linking RAAS hyperactivity to adipose tissue dysfunction, inflammation, and insulin resistance (contribution 4). This emphasizes the role of systemic RAAS overactivation in metabolic syndrome pathogenesis even prior to overt diabetes [8].

In the fifth contribution to this Special Issue, McGrath and Wentworth highlight the dualistic role of RAAS in hepatic pathology, where classical Ang II–AT1 signaling promotes fibrosis, inflammation, and portal hypertension, while the protective ACE2–Ang-(1–7)–Mas axis counteracts these effects (contribution 5). This knowledge reinforces the concept that RAAS is a master regulator of organ remodeling and disease progression beyond the cardiovascular system [9].

In the final contribution to this Special Issue, Tain and Hsu provide a comprehensive overview of RAAS in early-life programming, demonstrating that perinatal environmental insults dysregulate RAAS, predisposing individuals to hypertension, kidney disease, and metabolic disorders in adulthood (contribution 6). Epigenetic modifications, oxidative stress, and sex-specific vulnerabilities underscore RAAS as a developmental determinant of long-term cardiometabolic health.

Collectively, RAAS is not merely a hormonal cascade for blood pressure regulation but a systemic, organ-specific network orchestrating inflammation, metabolic dysfunction, and tissue remodeling across multiple organ systems. The dysregulation of RAAS in early life or in metabolic disease states propagates a cascade of cellular and molecular alterations, linking vascular, renal, hepatic, and adipose pathologies.

There are several clinical and translational implications associated with these studies. APA inhibitors, MR antagonists, and ACE2–Ang-(1–7) axis activators may provide organ-specific therapeutic benefits. Early-life dietary strategies or isoflavone supplementation could reprogram RAAS activity and prevent disease onset. Monitoring tissue-specific RAAS biomarkers (e.g., urinary angiotensinogen, central Ang III levels) may guide individualized therapeutic strategies [9].

In conclusion, the emerging evidence positions RAAS as a central integrator of multi-organ disease, linking early-life programming, vascular inflammation, metabolic dysfunction, and organ-specific remodeling. Understanding and targeting RAAS at multiple levels holds the potential to transform therapeutic approaches for hypertension, diabetic nephropathy, fatty liver disease, and the broader spectrum of cardiometabolic disorders.

## Figures and Tables

**Figure 1:**
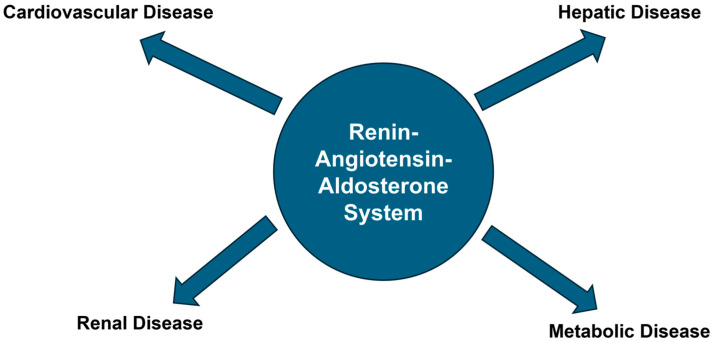
The renin–angiotensin–aldosterone system contributes a common pathway connecting cardiometabolic, renal, and hepatic disease.
